# The experience of fear and psychological distress among migrants in Spain

**DOI:** 10.3389/fpsyg.2025.1628841

**Published:** 2025-08-01

**Authors:** Milton Alexander López-de-León, Nina Hansen, Sabine Otten, Susana Puertas Valdeiglesias

**Affiliations:** ^1^Institute for Migration Research, University of Granada, Granada, Spain; ^2^Department of Social Psychology, University of Groningen, Groningen, Netherlands

**Keywords:** migrants, mental health, psychological distress, fear, risk factors, sociodemographic factors, immigration status, employment

## Abstract

**Objectives:**

Psychological distress is an unpleasant state with both emotional and psychological manifestations. Migrants are prone to facing psychological distress. Previous research investigated the risk factors migrants face when integrating in a new society. However, little attention was paid to the role of experienced fear. Therefore, this study aimed to investigate the prevalence of psychological distress of migrants and its link with the number of situations migrants may have experienced fear during and after the migration journey in addition to previously identified risk factors among migrants in Spain.

**Methods:**

381 migrants from different nations participated in a correlational study. Besides demographic information, we assessed four self-reported risk factors (immigration status, employment, social network, and Spanish proficiency). The relevant number of situations in which migrants experienced fear was assessed with four items (sum score) and psychological distress with the K10 Kessler Psychological Distress Scale. Important to note, the K10 did not include items assessing fear.

**Results:**

31.3% of migrants experienced severe levels of psychological distress. Three risk factors (immigration status, employment, and social network) and the sum score of fear-experienced situations correlated with psychological distress. A step-wise regression analysis (based on 10,000 Bootstrap samples) accounts for 15% of the variance in psychological distress, suggesting that (1) age (but not gender), (2) none of the risk factors, but (3) the sum score of fear-experienced situations showed a significant effect on psychological distress. Importantly, we assessed the amount of fear triggering situations migrants may have experienced (in contrast to general anxiety which is often measured and included in psychological distress scales).

**Conclusions:**

The current study provides first evidence that various situations in which migrants experienced fear during and after their journey to Spain were associated with higher levels of psychological distress; more strongly than previously studied risk factors. We discuss the importance of differentiating between past fear to prevent trauma and current fear (e.g., of mafias and of feeling sick) in reception centers to help migrants navigate the new cultural context. Focusing on the mental health needs of migrants after arrival could be a promising first step to stimulate integration more sustainably.

## 1 Introduction

117.3 million people worldwide were forcibly displaced at the end of 2023 (United Nations High Commissioner for Refugees, 2024). They had to flee because of conflict, violence, and human rights violations. Additionally, a total of 167,366 applications for international protection were submitted in 2024 (Ministerio del Interior, 2025). Many migrants^1^ from different cultures suffer from high levels of psychological distress that is caused by different experiences, e.g., dangerous border crossing or limited resources (Garcini et al., 2016). Migrants are more likely to experience higher levels of psychological distress (Thela et al., 2017), even more so than host society members (Georgiadou et al., 2017; Levecque et al., 2009). Previous research has identified and studied relevant risk factors in the current living context such as economic hardship, physical harm, poor living conditions, and social isolation which are related to the prevalence of mental disorders and deterioration of mental health (Chen et al., 2021; Giacco et al., 2018).

In addition to psychological distress, many migrants experience fear in a variety of situations during their migration journey. Also, after leaving the home country, fear may play a role in their daily lives in a country where they represent a minority. Migrants find themselves in difficult circumstances that often times force them to leave their countries, travel on dangerous routes, and face unexpected and new experiences (Dempsey, 2020; Moya and Puertas, 2008). The current research sets out to investigate the role of fear and its link to psychological distress in addition to relevant risk factors when they try to integrate into the new society.

## 2 Literature review and hypotheses

### 2.1 Psychological distress

Migrants face different challenges during their migration journey (Cohen et al., 2019). As a result, many migrants experience high levels of psychological distress (Cénat et al., 2020; García-Sierra et al., 2020). Psychological distress is defined as a set of painful mental and physical symptoms that are associated with normal fluctuations of mood in most people (American Psychiatric Association, 2013). Similarly, it has been described as unpleasant subjective state of depression and anxiety, which has both emotional and psychological expressions (Mirowsky and Ross, 2003). It can be experienced in response to a specific stressor or demand that causes harm, either temporary or permanent (Ridner, 2004). Migrants themselves express their symptoms with feelings of frustration, chest pain (Kerbage et al., 2020), tiredness, irritability, and social isolation (Tahir et al., 2022). In the current research, we assessed psychological distress with the Kessler Psychological Distress Scale (K10, Kessler et al., 2003). In line with previous research, we studied psychological distress as a transient (not long-lasting) phenomenon that is related with factors specific to the migration process.

Importantly, not every migrant will experience high level of psychological distress (e.g., Jurado et al., 2017; López-de-León and Puertas, 2022; Vromans et al., 2021). Below we will review previous research on relevant *demographic differences* and *risk factors*.

### 2.2 Demographic differences

The experience of psychological distress among migrants can differ considerably between sub-groups. In the current research, we focused on two important demographic differences, namely age and gender. Interestingly, the findings about both variables show mixed results. Regarding age, there is evidence that younger migrants show less emotional stress compared to older adults (e.g., Mekki-Berrada, 2018). Yet, in other studies, older people reported fewer depressive symptoms than younger people (e.g., Wu and Schimmele, 2005).

Similarly, several studies have found that women experienced higher levels of psychological distress than men (Hovey and King, 1996; Jarallah and Baxter, 2019), but other studies did not find gender differences with respect to anxiety, depression, post-traumatic stress, and suicide risk (e.g., Georgiadou et al., 2017). Accordingly, we did not hypothesize specific effects for age and gender but controlled for their potential impact in our analyses (in line with general principles for reducing bias; American Psychological Association, 2020).

### 2.3 Risk factors related to psychological distress

So-called risk factors may make migrants more vulnerable for mental health problems than others. We reviewed previous research and identified four relevant factors which we could also test in the current research. First, migrants who were still waiting for a response of their asylum request showed more mental health problems compared to migrants who had received a residence permit (Leiler et al., 2019) and were able to stay in the new host society (for similar results see Brietzke and Perreira, 2017; Cadenas et al., 2024; Patler and Pirtle, 2018; Pérez and Fortuna, 2005). Based on this research we expected that migrants who did not (yet) have a residence permit or were still unsure whether they would be allowed to stay should show higher levels of psychological distress compared to migrants who received a residence permit. We refer to this risk factor as *immigration status*.

Second, three studies conducted in Western European countries reported that migrants who did not have a job showed more psychological distress (Gómez-Salgado et al., 2024), specifically mood and anxiety disorders (Bogic et al., 2012; Suárez-Hernández et al., 2011). Observing these results, we assumed in our study that people who were unemployed should also show higher levels of psychological distress. We name this risk factor *employment*. Third, migrants who were in the process of adaptation and did not have a social network in the host country reported being depressed and exhausted (Hardi, 2005). Based on this and other similar studies (see Gerritsen et al., 2006; Schweitzer et al., 2006), we expected that migrants who did not have a *social network*, compared to those who had friends or compatriots in the reception country, should report greater symptoms of psychological distress.

Finally, the results of a study in Australia (Chou, 2007) reported that migrants' language barriers may be one of the reasons why migrants from Asian countries showed higher level of psychological distress (Buchanan et al., 2018). Therefore, we assumed that people who experienced difficulties with speaking the language (Spanish) needed to communicate in the host country typically also showed deterioration in their mental health. We refer to this risk factor as *Spanish proficiency*. In addition to the above factors, very little is known about the relation of emotional states, such as fear people experience in the different steps on the migratory process, and their link to migrants' psychological distress.

### 2.4 The role of fear (vs. psychological distress)

Measures of psychological distress assess it as a global measure tapping into feelings of such as unease, depression, unrest, and exhaustion (e.g., Kessler et al., 2003). Several scales include general feelings of anxiety. However, the scale we employed in the current study did not include items assessing fear. This is why we focused on specific feelings of fear during the migration process as well. Though certainly related, feelings of anxiety and especially fear can be considered to be more clearly linked to specific situations (such as having to travel under dangerous circumstances). That such specific situations related to fear are relevant and displaced populations may experience them differently has been recently recognized in research (Cohen, 2023; Lambert et al., 2024). Migrants face many unforeseen and dangerous situations during their migration journey and upon arrival that may trigger fear (for an overview see Dempsey, 2020; Laban et al., 2004). Fear is defined as a rapid emotional response to a clearly identifiable and specific threat (American Psychological Association, 2018; Davis et al., 2010). In contrast, anxiety is a response to potential, more diffuse threats, linked to a long-lasting state of arousal and vigilance.

The fear experienced throughout the migration process is evident both during the journey and upon arrival in the host country. For instance, migrants reported being afraid of being raped (Cohen et al., 2019), and attacked and suffered violence during their transit (Cénat et al., 2020). Besides, they reported being strongly fearful and horrified by the conditions of the journey, knowing that they could die at any moment (Brigden and Mainwaring, 2016). Research conducted with migrant populations that have arrived in a new host country has focused on additional aspects, which may trigger fear, such as the fear of repatriation to their home countries (Posselt et al., 2020; Sinnerbrink et al., 1997; Torres and Wallace, 2013), and the fear and worries about family in the home country (Nickerson et al., 2010). In the current research, we focused on specific aspects of fear which migrants may experience themselves (and not the fear they may have for their family members).

Research has highlighted that migrants, on average, neither experience more violent events in host countries (Ben Farhat et al., 2018) nor are more afraid (Castañeda et al., 2021; Hedstrom et al., 2021) than they experienced before they started to migrate. However, some migrants experienced violence in host countries, which actually surpassed what they had experienced in other geopolitical environments (Dempsey, 2020; Stodolska and Shinew, 2009). Despite low rates of criminal organizations in European countries, migrants are still at risk of being threatened by mafias or caught up in a human trafficking network. To date, research has mainly focused on migrants' fear if they need to seek medical help, but have an undocumented immigrant status (Alsharif, 2022; Bahar-Özvariş et al., 2020; Chuah et al., 2018; Teunissen et al., 2014), and of misjudgment by doctors (Feldmann et al., 2007; Liebling et al., 2014). Lastly, migrants mentioned that they were afraid of dying once they settled (Eleftherakos et al., 2018), also because the new social context was dangerous (Vives-Cases et al., 2008).

To conclude, migrants may face unforeseen and dangerous situations during their migration journey, and despite finally having arrived in a country that can be considered “safe,” they may still face situations triggering fear. To date, research in this field mostly relied on qualitative insights (e.g., Chuah et al., 2018; Lambert et al., 2024; Teunissen et al., 2014). In the current quantitative study among migrants living in Spain, we set out to study in how far variations in migrants' level of distress can be explained by their experiences of fear in specific situations (fear during the journey, of mafias, of getting sick, and of dying) and by previously studied risk factors.

### 2.5 The current study

The ultimate aim of the present research was to investigate the prevalence of psychological distress of migrants and its link with experienced fear during and after the migration journey in addition to previously identified risk factors. First, we expected that migrants would experience high levels of psychological distress. Second, we hypothesized that (1) risk factors and migrants' fear would be positively associated with higher levels of psychological distress, and (2) that the link between fear and psychological distress will persist even when controlling for risk factors (i.e., immigration status, employment, social network, and Spanish proficiency). To test our hypotheses, we collaborated with reception centers and non-governmental organizations in Spain to be able to survey migrants from different host societies who were in different stages of their integration process in Spain.

## 3 Materials and method

### 3.1 Positioning and reflectivity statement

The first author is a migrant himself and participated as a volunteer in different non-governmental organizations. This helped to encourage migrants to participate in the study. The second, third, and fourth authors of this article were born in European countries and belong to ethnic majority groups in Germany and Spain.

### 3.2 Collaboration and preparation of research materials

The first author approached different organizations and shared a research proposal with them. He organized several meetings to gain trust and receive support of public and private institutions. Coordinators of seven migrant reception projects gave permission to approach and invite migrants to fill in the questionnaire during the activities in the reception centers.

The questionnaire was carefully adjusted to the target group in Spanish. The questionnaire was pilot tested with 20 migrants with the support of two French and Punjabi translators. Afterwards, the questionnaire in Spanish was translated to English, French, Arabic, and Russian (see model of the process of translation and cultural adaptation (Ortiz-Gutiérrez and Cruz-Avelar, 2018): preparation, forward translation, integration, back-translation review, drafting, user testing, finalization, and final report). If needed, native translators helped during the data collection if people were lowly educated and had difficulties in understanding the questions or spoke another language.

### 3.3 Study design and sample

This study had a correlational design. For further research and replication, you can access the public database and coding at the Supplementary material. In total, 381 migrants (47.4% women and 52.6% men) participated in the study. All participants were living in and around Valencia, Spain, at the time of the study and were on average 36.65 years old (*SD* = 11.51, range 18–74 years). They came from 51 different countries, mainly in Africa, Latin America, Middle East, Eastern Europe, and South-East Asia. The largest group in our sample came from Colombia (15.7%) followed by migrants from Venezuela (10.8%), Morocco (10.5%), Algeria (7.3%), Russia (5.8%), Ukraine (5.2%), Honduras (4.5%), and Nigeria (4.5%). Unfortunately, the subsamples were too small to conduct group comparisons. The majority of participants were staying in Spain for less than a year (44.3%), or 1 year (15.3%); 8.7% for 2 years, less for 3 years (4%), 4 and 5 years (each 2.1%), and 23.5 % stayed already more than 5 years in Spain. A very small number (1.3%) of the participants had no schooling, or only primary schooling (6.8%); most had followed secondary education and/or vocational training (53.7%) and 38.2% had followed university education.

### 3.4 Procedure

The research was approved by the academic committee of the University of Granada. The first author used convenience and snowball sampling to invite a diverse group of migrants in the Valencian Community, in Spain, for 1 year. All respondents were invited to participate in a study on mental health.

Participants were orally informed about the study aim and content by the first author individually or in groups and could also read the informed consent. After giving oral consent migrants received the questionnaire in the language they preferred (English, French, Russian, Spanish, and Arabic). They filled in the questionnaire alone or with the help of a native speaker if needed. Participants answered questions about their demographic background, fear, and psychological distress. All participants joined voluntarily and were thanked for their participation.

### 3.5 Measures

#### 3.5.1 Demographic variables

We asked participants to indicate their age, gender, country of origin, educational attainment, and length of stay (see sample description).

#### 3.5.2 Risk factors

We assessed risk factors with four items and variables were coded such that higher scores imply higher risk (all self-reported). Participants were asked to indicate their *immigration status* with one item with four response options: undocumented migrant, asylum seeker, refugee, or documented migrant/European citizenship. We recoded the four answer options in a dichotomous categorical variable with 1 for documented migrants (documented migrants) vs. 2 for undocumented migrants/asylum seekers (undocumented migrants, asylum seekers, and refugees). Next, we assessed migrants' *employment* status with one item offering five response options: jobless, temporary or permanent job, freelancer, or does no need to work. We recoded the answers into a dichotomous categorical variable with 1 for employed/no need to work (temporary or permanent job, freelancer, and does no need to work) vs. 2 for unemployed (jobless). To assess migrants' *social network*, we asked a single question (e.g., De la Revilla et al., 2011) whether they had compatriots or friends with whom they identified themselves in Spain (answer options were 1 for yes and 2 for no). Next, we assessed migrants' *Spanish proficiency* with one item measuring their difficulties to communicate in Spanish with three answer options: 1 for no difficulties, 2 for medium difficulties, or 3 for high difficulties.

#### 3.5.3 Fear

We assessed the number of situations in which migrants experienced fear with four items which we identified based on a literature review (e.g., De la Revilla et al., 2011). All participants were asked whether or not (coded 0 for no, 1 for yes) they experienced fear in specific situations during their migration journey and after arriving in Spain: (1) Have you been afraid on your trip to Spain?, (2) Are you afraid of the mafias/gangsters in Spain?, (3) Are you afraid to get sick and that they do not know how to heal you in Spain?, and (4) Are you afraid of dying in Spain?. We created a sum score to assess the frequency of experienced fear (from 0 for *no fear at all* to 4 for *a lot of fear situations*). Inspection of the inter-item correlations showed that all items were significantly correlated, all *r*'s > 0.14, *p*'s < 0.01. However, the reliability was low (α = 0.60). This is not that surprising given the dichotomous nature of the four items (McNeish, 2024; Sijtsma et al., 2024).

#### 3.5.4 Psychological distress

We assessed psychological distress with the adaptation of the K10 Kessler Psychological Distress Scale (Kessler et al., 2003) for Spanish (Lisis Group, 2011). Migrants were asked to answer 10 questions about how they felt in the last month (with five response options: 1 for *never*, 2 for *rarely*, 3 for *sometimes*, 4 for *many times*, and 5 for *always*; example item: How often have you felt tired for no good reason?). We used two indicators: (1) classification in prevalence and (2) a mean score. First, the total score for distress is determined by adding up the answers on all 10 items (with a theoretical range from 10 to 50). Next, in line with Kessler et al. (2003), the scores were classified into four categories: *no psychological distress* (score between 10 and 19), *mild psychological distress* (between 20 and 24), *moderate psychological distress* (between 25 and 29), and *severe psychological distress* (between 30 and 50). Second, we computed a mean score using the actual score per participant for our regression analyses testing the hypotheses; the scale showed high reliability (α = 0.88).

## 4 Results

### 4.1 Descriptive results

See Table 1 for an overview of the key study variables. 38.5% of the surveyed participants were documented migrants whereas 61.5% were undocumented and asylum seekers. Furthermore, 26.2% of the respondents had a job (or did not need to work) while the majority of the migrants (73.8%) were unemployed at the time of the survey. Most respondents indicated having a social network (73.4%) compared to 26.6% who did not have. In addition, most migrants indicated having no difficulties with communicating in Spanish (57.4%), while others indicated medium difficulties (32.6%), and a small group did not speak Spanish at all (10%).

**Table 1:** Overview of the descriptive statistics of all investigated variables.

**Variables**	**% (*n*)**	***M* (*SD*)**
**Immigration status**
Documented migrants	38.5% (145)	
Undocumented migrants/Asylum seekers	61.5% (232)	
**Employment**
Employed/No need to work	26.2% (100)	
Unemployed	73.8% (281)	
**Social network**
Yes	73.4% (278)	
No	26.6% (101)	
**Spanish proficiency**
No difficulties	57.4% (218)	
Medium difficulties	32.6% (124)	
High difficulties	10% (38)	
**Level of psychological distress**
No psychological distress	25.1% (90)	
Mild psychological distress	21.5% (77)	
Moderate psychological distress	22.1% (79)	
Severe psychological distress	31.3% (112)	
Fear		1.68 (1.3)
Psychological distress		2.57 (0.80)

Looking at the clinical classification, 25.1% of the respondents classified as having no psychological distress (scoring under 19) while 21.5% experienced mild (scoring between 20–24), 22.1% moderate (scoring between 25–29), and 31.3% even severe levels of psychological distress (scoring over 30). The average level of migrants' experienced psychological distress falls between rarely and sometimes (*M* = 2.57, *SD* = 0.80). We used this mean score for the correlational and regression analyses below. Important to note, migrants on average experienced 1.68 situations which triggered fear (*SD* = 1.3). Inspection of the frequency of each item showed that people differed in the situations they experienced fear; the most frequent situation in which they experienced fear was fear of mafias (46.2%). In addition, 44.9% of the participants experienced fear during the journey, 44.4% experienced fear of getting sick and not being healed in Spain, and 32.5% of the participants, fear of dying in Spain.

### 4.2 Main analyses

According to the normality analysis through Kolmogorov-Smirnov test *K*−*S*_(347)_ = 0.058, *p* < 0.01, the residuals had a non-normal distribution. Therefore, Spearman non-parametric tests were used. As we expected (hypothesis 1), fear correlated positively with psychological distress (*r* = 0.34, *p* < 0.001). Moreover, age correlated negatively with psychological distress (*r* = −0.21, *p* < 0.001) while gender did it positively with fear (*r* = 0.14, *p* < 0.01). In addition, immigration status correlated positively with both psychological distress (*r* = 0.17, *p* < 0.01) and fear (*r* = 0.13, *p* < 0.05). Furthermore, employment correlated positively with psychological distress (*r* = 0.15, *p* < 0.01) and fear (*r* = 0.15, *p* < 0.01). Finally, social network correlated positively with psychological distress (*r* = 0.11, *p* < 0.05) but not fear (see all correlations in Table 2).

**Table 2:** Overview of means, standard deviations (where applicable), and correlations of all study variables.

**Variables**	***M* (*SD*)**	**1**.	**2**.	**3**.	**4**.	**5**.	**6**.	**7**.	**8**.
1. PD (1–5)	2.57 (0.80)	–	−0.21^***^	0.03	0.17^**^	0.15^**^	0.11^*^	0.02	0.34^***^
2. Age			–	0.19^***^	−0.34^***^	−0.15^**^	−0.14^**^	−0.11^*^	−0.05
3. Gender				–	−0.22^***^	0.06	−0.05	−0.03	0.14^**^
4. IS (1–2)					–	0.28^***^	0.18^***^	0.15^**^	0.13^*^
5. EM (1–2)						–	0.12^*^	0.08	0.15^**^
6. SN (1–2)							–	0.16^**^	0.02
7. SP (1–3)								–	−0.05
8. Fear (0–4)	1.68 (1.3)								–

PD, psychological distress; IS, immigration status; EM, employment; SN, social network; SP, Spanish proficiency.

^***^p < 0.001, ^**^p < 0.01, ^*^p < 0.05.

Next, we test hypothesis 2 with a stepwise regression analysis based on 10,000 Bootstrap samples with psychological distress as the outcome variable. In step 1 we controlled for the impact of age and gender. This model explained 4% of the variance in psychological distress [*R*^2^ = 0.04, *F*_(2.344)_ = 6.90, *p* = 0.001]. More specifically, gender did not significantly explain variance [*B* = 0.11, *SE* = −0.00, *BCa 95% IC* (−0.07, 0.27), *p* = 0.233], whereas age did [*B* = −0.01, *SE* = 0.00, *BCa 95% IC* (−0.02, −0.01), *p* < 0.001]: older respondents reported less psychological distress than younger respondents. In the second step, the potentially risk factors variables were included. This model explains 6% of the variance [*R*^2^ = 0.06, *F*_(6.340)_ = 3.90, *p* < 0.001]; age still explains a significant amount of variance [*B* = −0.01, *SE* = 0.00, *BCa 95% IC* (−0.02, −0.00), *p* = 0.007]. However, surprisingly, the potential risk factors immigration status, employment, social network, and Spanish proficiency did not significantly add to the variance explained in psychological distress. In step 3, when fear was added, this model explained a substantially higher amount, namely 15% of the variance [*R*^2^ = 0.15, *F*_(7.339)_ = 8.84, *p* < 0.001]. Albeit somewhat weaker age had a significant effect on psychological distress [*B* = −0.01, *BCa 95% IC* (−0.02, −0.00), *p* = 0.011]. Importantly the combined score for fear was a significant predictor of psychological distress [*B* = 0.19, *SE* = 0.00, *BCa 95% IC* (0.13, 0.25), *p* < 0.001] stronger than the demographic variables and risk factors (see all steps in Table 3).

**Table 3:** Results of stepwise regression analysis for psychological distress (*N* = 347).

**Model**	***B***	**(SE)**	**95% CI Lower bound**	**95% CI Upper bound**	***p***	***R^2^***	**ΔR^2^**	***F* (o χ^2^)**
**Step 1: Control variables**
Age	−0.01	(0.00)	−0.02	−0.01	<0.001	0.039	–	6.895^**^
Gender	0.11	(−0.00)	−0.07	0.27	0.233			
**Step 2: Risk factors**
Age	−0.01	(0.00)	−0.02	−0.00	0.007	0.064	0.025	3.896^***^
Gender	0.12	(−0.00)	−0.05	0.29	0.179			
Immigration status	0.16	(0.00)	−0.02	0.34	0.085			
Employment	0.14	(−0.00)	−0.06	0.34	0.162			
Social network	0.13	(0.00)	−0.07	0.33	0.211			
Spanish proficiency	−0.05	(−0.00)	−0.18	0.07	0.434			
**Step 3: Fear**
Age	−0.01	(0.00)	−0.02	−0.00	0.011	0.154	0.090	8.841^***^
Gender	0.03	(−0.00)	−0.14	0.19	0.762			
Immigration status	0.09	(0.00)	−0.09	0.27	0.334			
Employment	0.09	(0.00)	−0.10	0.28	0.335			
Social network	0.13	(0.00)	−0.06	0.32	0.185			
Spanish proficiency	−0.01	(0.00)	−0.14	0.11	0.852			
Fear	0.19	(0.00)	0.13	0.25	<0.001			

Standardized coefficients. ^**^p < 0.01; ^***^p < 0.001.

## 5 Discussion

The central aim of the current study was to investigate the prevalence of experiencing psychological distress among migrants in Spain and its links with experienced fear in addition to relevant risk factors. Almost a third of the migrants in our study reported severe levels of psychological distress. This prevalence is comparable with other results obtained in Spain (García-Sierra et al., 2020; Gómez-Salgado et al., 2024) and other countries (Cénat et al., 2020; Thela et al., 2017).

As expected, except language fluency, risk factors identified in the literature (immigration status, employment, and social network), and fear were positively correlated with psychological distress. Yet, when testing our hypothesis in a stepwise regression analysis we did not find any significant links between the risk factors and psychological distress. In this analysis, except a significant effect of migrants' age, the number of situations in which migrants experienced fear was strongly associated with psychological distress. This result suggests that focusing on fear in addition to the known risk factors could be an important step to gain a deeper understanding of the triggering factors of psychological distress. In fact, the present results suggest that addressing migrants' fear experiences might be an especially promising avenue to enhance their mental wellbeing and, relatedly, their functioning in the host country.

The current study zoomed in on a number of specific fear experiences that migrants may experience. The choice of this list of potential fear experiences was based on insights from previous qualitative research (Bracken and Gorst-Unsworth, 1991; Schockaert et al., 2020; Stodolska and Shinew, 2009). Yet, the present data can obviously only offer a first insight in the role of fear among migrants, and will hopefully inspire further research.

While the correlation analysis showed significant links between psychological distress and the three investigated risk factors, these links became insignificant when entered simultaneously in the stepwise bootstrapped regression analysis. This was unexpected, given that multiple studies have reported a significant impact of these risk factors on the migrants' mental health (Bogic et al., 2012; Brietzke and Perreira, 2017; Cadenas et al., 2024; Chen et al., 2021; Chou, 2007; Gerritsen et al., 2006; Jurado et al., 2017; Schweitzer et al., 2006). At the moment, we can only speculate about the reasons. Possibly, the simplistic operationalization of the risk measures, and/or the fact that on several risk factors the respondents scored predominantly low (especially regarding having a social network and speaking Spanish) may have played a role here and, therefore, attenuate the observed associations in the regression analysis. Moreover, given that the correlations between risk factors and distress were significant, a large amount of shared variance together with the already mentioned floor effect could account for this finding. On the other hand, and more importantly, the stepwise regression analysis indicated that experiences of fear may be more relevant for migrants' distress than the recently studied risk factors. These results offer a preliminary hypothesis of how and which situations in which migrants experienced fear may have an effect on the mental health of migrants.

### 5.1 Practical implications

In our view, the current results offer one clear practical implication. Some migrants may develop more severe forms of PTSD or anxiety and depression that would require specialized support. However, many migrants may not need this clinical support. Rather, public reception centers or non-governmental organizations could try to offer psychosocial help to help migrants handle past experiences of fear and potentially help avoiding fear-triggering situations in the new host society. For example, by differentiating between past fear to prevent trauma and current fear (e.g., of mafias and of getting sick), to help them navigate in the new cultural context; and by explaining to migrants how to detect trafficking networks, or gangsters, as well as the recruitment process of these groups, and informing them about their rights and services with respect to health care, migrants' distress could decrease. This psychosocial support should be offered besides practical support (e.g., housing, employment) and helping migrants to develop a new network (e.g., with providing buddies from the host society; Heikamp et al., 2025).

### 5.2 Limitations and future research

In our view, this study has three main limitations. First, the correlational design does not allow us to draw any causal conclusions. Based on previous research, we assume that the number of situations in which migrants experienced fear may trigger psychological distress. However, we cannot rule out that psychological distress that many migrants experience may also increase the likelihood that they may experience fear in several unforeseen new situations during their migration journey. In this scenario, symptoms of depression and anxiety may affect selective attention, leading the person to focus more on threatening stimuli, thus reinforcing their sense of insecurity. Similarly, anxiety symptoms may lead to a state of hypervigilance, where ambiguous interactions might be interpreted as danger signals (see Cohodes et al., 2021). Therefore, the use of a longitudinal or multilevel design and/or a mixed-method approach linking qualitative and quantitative data could help to determine the direction of the effect. Most importantly, these designs would provide stronger grounds for causal interpretation and a better understanding of contextual influences.

Second, a potential selection bias (recruitment via the social network of the first author), small subsamples of different ethnic groups, and the skewed distribution across potential risk factors may have influenced the present findings. Unfortunately, the study had insufficient statistical power to conduct additional subgroup analyses (e.g., region of origin and educational attainment). A more representative, or a more homogeneous sample (e.g., people from the same country of origin) may offer more systematic insights on how and which risk factors, together with the number of situations in which migrants experience fear, may have a negative impact on migrant's mental health.

Third, we only assessed a limited number of situations in which migrants experienced fear with a sum score. Future research should assess fear more systematically, covering a broader range of potentially fear-eliciting situations (e.g., fear migrants may experience because of the situation of family members, and fear due to discrimination). In addition, future research should employ psychometrically validated instruments to capture the intensity of fear (and the other structural disadvantages) more comprehensively. Recent research conducted among Syrian refugees showed that especially living in displacement triggered fears about the family (how they are doing, whether they could see each other again) and the future of their children (Lambert et al., 2024).

## 6 Conclusion

Experiencing fear during the migration process seems to be a frequent experience for migrants and was linked to higher levels of psychological distress beyond other risk factors. We offered some first evidence for the importance to better understand which type of fear triggering situations migrants may experience. Inclusive public policies and psychosocial support offered by for social workers or volunteers after migrants arrived in these countries could help migrants who want to start a new life and thrive in a new culture (see also Cobb et al., 2019)—without fear.

## Data Availability

The original contributions presented in the study are included in the article/Supplementary material, further inquiries can be directed to the corresponding author.

## Footnotes

^1^We will use the term migrant throughout the manuscript to include the different reasons why people had to flee.
