# Supplementary material for: The experience of fear and psychological distress among migrants in Spain
Source: Front Psychol. 2025 Aug 1;16:1628841. doi: 10.3389/fpsyg.2025.1628841 (PMC12354457; doi:10.3389/fpsyg.2025.1628841)
Supplement: Supplementary file 1 [file Supplementary_file_1.docx]

Supplementary Material

# Coding (Syntax)

*Normality

REGRESSION

/MISSING LISTWISE

/CRITERIA=PIN(.05) POUT(.10)

/NOORIGIN

/DEPENDENT MEAN_PD

/METHOD=ENTER Age Gender

/METHOD=ENTER Immig_Status Employment Social_netwo Spanish_prof

/METHOD=ENTER fear_SUM

/SAVE RESID.

EXAMINE VARIABLES=RES_1

/PLOT BOXPLOT NPPLOT

/COMPARE GROUPS

/STATISTICS DESCRIPTIVES

/CINTERVAL 95

/MISSING LISTWISE

/NOTOTAL.

*Correlation

NONPAR CORR

/VARIABLES=MEAN_PD Age Gender Immig_Status Employment Social_netwo Spanish_prof fear_SUM

/PRINT=SPEARMAN TWOTAIL NOSIG FULL

/MISSING=PAIRWISE

*Regression

BOOTSTRAP

/SAMPLING METHOD=SIMPLE

/VARIABLES TARGET=MEAN_PD INPUT= Age Gender Immig_Status Employment Social_netwo Spanish_prof

fear_SUM

/CRITERIA CILEVEL=95 CITYPE=PERCENTILE NSAMPLES=10000

/MISSING USERMISSING=EXCLUDE.

REGRESSION

/MISSING LISTWISE

/STATISTICS COEFF OUTS R ANOVA

/CRITERIA=PIN(.05) POUT(.10)

/NOORIGIN

/DEPENDENT MEAN_PD

/METHOD=ENTER Age Gender

/METHOD=ENTER Immig_Status Employment Social_netwo Spanish_prof

/METHOD=ENTER fear_SUM.
